# MPNST of the abdominal wall in a patient with lynch syndrome: A case report of a rare presentation and unique association

**DOI:** 10.1016/j.ijscr.2023.108677

**Published:** 2023-08-18

**Authors:** Anis Hasnaoui, Salma Kacem, Imen Sassi, Fakhreddine Ben Abdallah, Sondes El Guesmi

**Affiliations:** a Faculty of Medicine of Tunis, Tunis El Manar University, Rue Djebal Lakhdar, 1006 Tunis, Tunisia; b Department of General Surgery, Menzel Bourguiba Hospital, Tunisia; c Department of Oncologic Surgery, Salah Azaiez institute, Tunis, Tunisia; d Department of Pathology, Menzel Bourguiba Hospital, Menzel Bourguiba, 7050 Bizerte, Tunisia; e Department of Oncology, Menzel Bourguiba Hospital, Menzel Bourguiba, 7050 Bizerte, Tunisia

**Keywords:** Sarcoma, Malignant peripheral nerve sheath tumors, Lynch syndrome, MLH1, Case report

## Abstract

**Introduction:**

The abdominal wall is an extremely rare location for malignant peripheral nerve sheath tumors (MPNSTs). Besides presenting a rare location of MPNST, the peculiarity of our case lies in its association with Lynch syndrome, which is to our knowledge the first reported case of its kind.

**Presentation of case:**

We present a case report of a 39-year-old male with a personal history of colonic cancer. Genetic counseling revealed Lynch syndrome with a heterozygous germline mutation in MLH1. Nine years after the right hemicolectomy, the patient presented with an asymptomatic lump in the abdominal wall. CT imaging showed a 3 cm mass in the aponeurosis of the right external oblique muscle. The patient underwent successful resection of the parietal tumor. Pathological examination revealed an MPNST. No additional treatment was warranted, and the patient exhibited no signs of relapse during the six months following the surgery.

**Discussion:**

MPNSTs of the anterior abdominal wall are extremely rare and challenging. Some studies have investigated the presence of mismatch repair (MMR) deficiency in patients with sarcomas. Our case consolidates the hypothesis of an association between sarcomas and Lynch syndrome, which raises the question of the efficacy of immune checkpoint inhibitor therapy in these cases where treatment options remain limited.

**Conclusion:**

It is essential to have a deep understanding of the growth patterns of MPNSTs in the context of syndromes that predispose individuals to tumors, like Lynch syndrome. This knowledge is crucial for accurately predicting patient outcomes and developing appropriate plans for monitoring and treatment.

## Highlights

• The abdominal wall is a rare location of MPNST.
• First reported association of an MPNST with Lynch syndrome
• Sarcomas could be a rare manifestation of mismatch repair deficiency.

## 1 Introduction

Schwannomas are usually benign tumors with rare cases of malignant transformation. Malignant schwannomas are now called malignant peripheral nerve sheath tumors (MPNSTs) [1]. The term “malignant schwannoma” has become outdated following the implementation of the new classification system introduced by the World Health Organization (WHO) in 2021 [2]. MPNSTs represent approximately 10 % of all soft tissue sarcomas and are found in 4 % of patients with neurofibromatosis type 1 [3]. MPNSTs most commonly occur in the deep soft tissues, usually close to a nerve trunk. The most common sites are the sciatic nerves, brachial plexus, and sacral plexus. The abdominal wall is an extremely rare location for these tumors. Primary abdominal wall sarcomas account for less than 5 % of cases [4]. Besides presenting a rare location of an MPNST, the peculiarity of our case lies in its association with Lynch syndrome, which is to our knowledge the first reported case of its kind. This work has been reported in line with the SCARE criteria [5].

## 2 Presentation of case

**Fig. 1:**
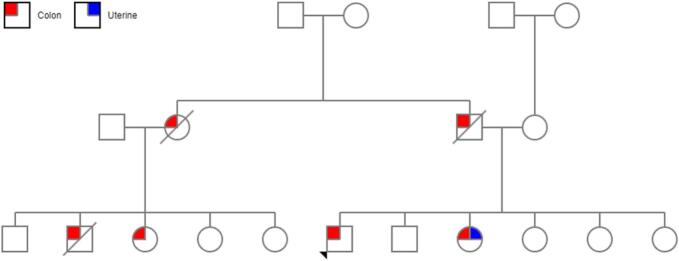
Family pedigree. Proband indicated with a black arrowhead.

We present a case report of a 39-year-old male with a family history fulfilling Amsterdam 2 criteria for clinical diagnosis of hereditary nonpolyposis colorectal cancer (Lynch syndrome) (Fig. 1), and personal history of colonic cancer.

In 2013, an acute onset of pain in the right iliac fossa accompanied by fever led to a diagnosis of acute appendicitis and subsequent emergency surgery. An infected cecal mass was discovered during laparotomy, leading to a carcinologic right colectomy. Pathological examination revealed a colloid carcinoma of the colon, classified as pT3N0M0. The patient underwent six months of adjuvant chemotherapy (FOLFOX) and remained under close observation. After 18 months, hepatic metastasis in the second segment was detected during imaging, resulting in a second laparotomy and a left lateral sectionectomy. The patient received an additional six months of FOLFOX and showed no further signs of systemic illness. Due to his young age and his family history, a genetic counseling was conducted, revealing Lynch syndrome with a heterozygous germline mutation in MLH1.

**Fig. 2:**
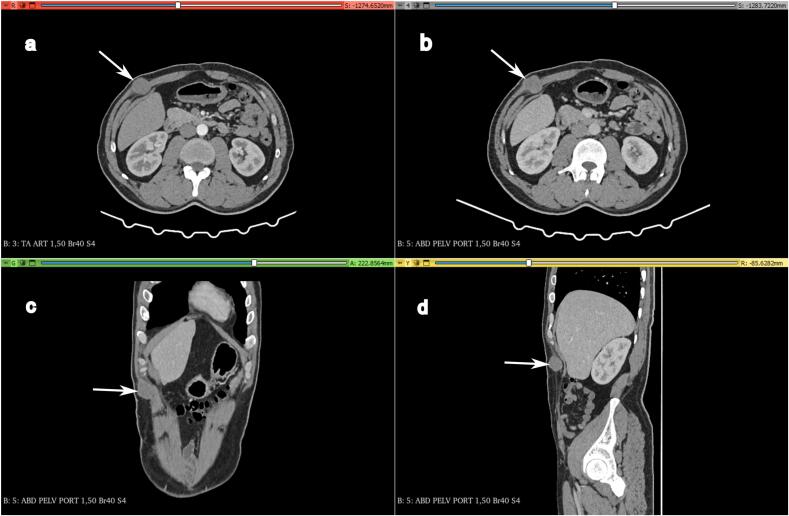
CT imaging showing the tumor (a) Arterial phase axial view and (b) portal phase axial view CT images showing no specific pattern of enhancement of the tumor (White arrow) (c) Coronal view and (d) sagittal view CT images revealing a 3 cm mass (White arrow) in the aponeurosis of the right external oblique muscle with well-defined contours.

**Fig. 3:**
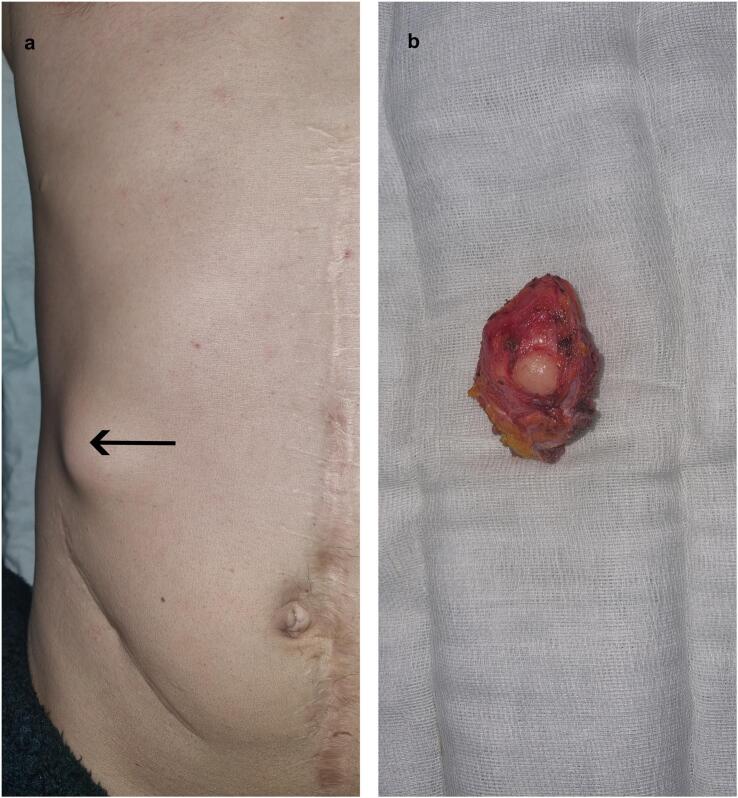
Preoperative and postoperative views of the tumor (a) Preoperative picture showing a three-centimeter lump in the right upper quadrant distant from any surgical scar (b) Postoperative specimen showing a well-limited nodule measuring 4*3.5 cm.

**Fig. 4:**
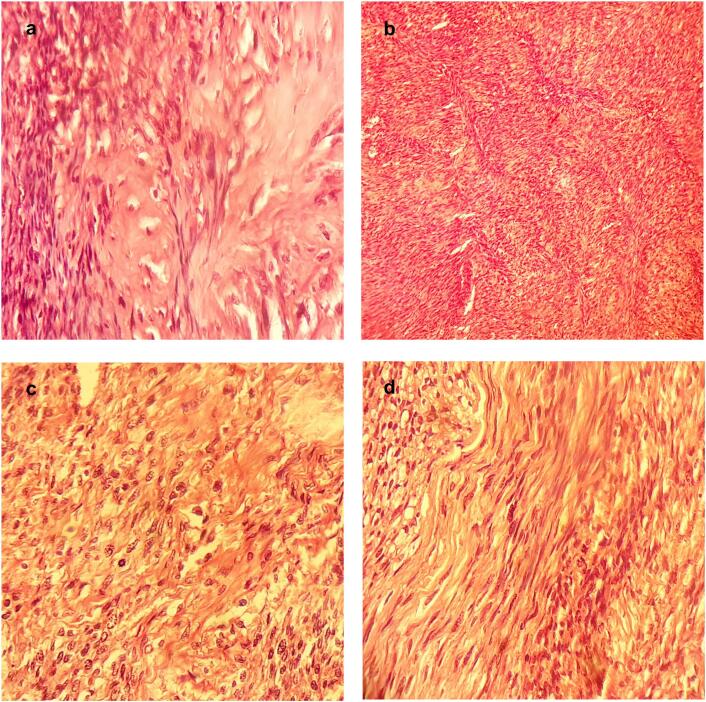
Histopathology images of the surgical specimen leading to the diagnosis of malignant peripheral nerve sheath tumor (a) Marbling Alternating tumoral hypercellular and hypocellular areas (x 100) (b) Fascicular connective tissue-like tumor proliferation (x 100) (c) Cytonuclear atypia with mitoses (x 400) (d) Wavy appearance of tumor cells (spindle cells) (x100).

In 2022, nine years after the right hemicolectomy, the patient presented with an asymptomatic lump in the abdominal wall. Upon clinical examination, a hard three-centimeter mass was found in the right upper quadrant distant from the surgical scars, and CT imaging revealed a 3 cm mass in the aponeurosis of the right external oblique muscle with well-defined contours and spontaneous tissue density (Fig. 2). This prompted further evaluation to determine whether the mass was a parietal relapse of the primary colic tumor. The patient underwent successful resection of the parietal tumor with all layers removed in depth until reaching the floating ribs, resulting in clear margins (Fig. 3). Pathological examination revealed a malignant peripheral nerve sheath tumor characterized by marbling (alternating areas of hypocellularity and hypercellularity), a fascicular connective tissue-like tumor proliferation, asymmetrical spindle cells, and cytonuclear atypia with mitoses (Fig. 4). Mitotic activity was present in up to 40/10 high power fields. The postoperative course was uneventful, and the patient was referred to another healthcare facility for potential radiotherapy. After a multidisciplinary discussion, it was determined that no additional treatment was warranted, and the patient exhibited no clinical or radiological signs of relapse during the six months following the surgery. Genetic counseling was recommended to all family members, but they refused to comply.

## 3 Discussion

Sporadic MPNSTs usually occur in patients with an age ranging from 30 to 50 years. When associated with neurofibromatosis type 1 (NF1), these tumors can manifest in younger patients [6,7]. Frequently, MPNST causes neurologic deficits in the distribution of the involved nerves due to impingement or mass effect [3]. Typically, this type of tumor affects the limbs. However, it may also occur in various other areas of the body, such as the head and neck, trunk, pelvis, retroperitoneum, mediastinum, and gastrointestinal tract. It is noteworthy that these tumors are exceedingly rare in parenchymal organs like the liver and pancreas [8]. MPNST of the anterior abdominal wall is also extremely rare and challenging. In fact, this location of soft tissue sarcomas may require extensive surgery. It implies a mandatory 1 mm of normal tissue between the tumor and the inked resection [9]. It is also believed that anatomical barriers limit the spread of these tumors and can be respected when not invaded. Sometimes even en-bloc resections (bone, intra-abdominal organs, inguinal ligament resections) with full-thickness abdominal wall resection cannot ensure clear margins of 1 mm but are not associated with higher local recurrence [10]. The abdominal defect is managed by direct closure, flaps, or the use of prosthetic mesh. The choice of the suitable technique depends on the tumor size and the possible complications. Direct closure can be preferred for tumors less than 8 cm. The use of prosthetic mesh is associated with a higher rate of postoperative complications since abdominal wall soft tissue tumor surgery tends to have a high risk of infection by itself [10].

The definitive confirmation of MPNST is based on the histologic examination. Macroscopically, tumors are usually larger than 5 cm with a white-gray firm to fleshy cut surface, necrotic areas, and hemorrhage. Histologically, the most specific features of MPNST are fascicles with alternating, marble-like cellularity, palisade/rosette-like arrangements, and asymmetric spindle cells [11]. New studies suggest the need to combine pathology with genomic and molecular techniques for a better differential diagnosis and classification of MPNST [12]. In MPNST, several tumor suppressor genes are commonly mutated, including NF1, CDKN2A, and components of the polycomb repressive complex 2 (PRC2), including SUZ12 and EED. TP53 is also frequently lost or mutated. Also, MPNST often shows recurrently altered chromosomal regions, particularly constituting somatic copy number gains [12,13].

Lately, some studies have investigated the presence of mismatch repair (MMR) deficiency in patients with sarcomas. There are experimental (in mice), epidemiologic, histopathological, and molecular data for supporting the hypothesis that sarcomas could be a rare manifestation of MMR deficiency [14]. In a molecular screening of 353 bone and 539 soft tissue tumors, MMR deficiency was detected in 1 % of the total cohort and in 3 % of MPNST. [15] Having MMR deficiency in the tumor cells does not necessarily indicate the presence of a Lynch syndrome. Genetic testing is mandatory to confirm this diagnosis. To our knowledge, this is the first reported case in the literature of MPNST associated to a Lynch syndrome. Unlike in Gardner syndrome, there is no known association with soft tissue sarcomas in Lynch syndrome. However, two other phenotypes of Lynch syndrome have been described: Muir-Torre syndrome, which is defined by an association with sebaceous tumors, and Turcot syndrome defined by an association with tumors of the central nervous system [16,17]. This unique association is of paramount importance. First, it reinforces the hypothesis of a potential association between sarcomas and Lynch syndrome as suggested by Dominguez-Valentin et al. [18], and consequently, patients with sarcomas should be tested for potential MMR deficiency. Second, it raises the question of the efficacy of immune checkpoint inhibitor therapy in these cases where treatment options remain limited [19].

On the other hand, some studies have investigated the link between MPNST and familial carcinologic history [7,20]. When comparing the family history of a group of sporadic MPNST and a group of NF1-associated MPNST, patients with sporadic MPNST had a significantly higher rate of positive family history of malignancy than patients from the second group. Within the overall MPNST group, 4.6 % had a positive first-degree family history of colon cancer. This suggests an underlying genetic predisposition to the formation of malignancies [7]. The risk of developing a second primary malignancy in patients with a past personal history of colorectal cancer is higher than that in the general population [21].

It's essential to delve deeper into the limitations in the existing published literature. MPNSTs may be underrepresented due to their rarity, which in turn restricts the depth of our current knowledge. This opens an intriguing opportunity to explore how we can expand our understanding of this entity. To address this gap, there's a potential avenue for enhancing the data landscape. This could involve the establishment of comprehensive data reporting mechanisms, allowing for the collection of information from a wider spectrum of cases. By pooling data from various sources, we could develop a more comprehensive and nuanced perspective on MPNSTs. the involvement of cancer registries could significantly amplify these efforts. Their role in systematically collecting and collating cancer-related data makes them a prime candidate for contributing to the understanding of rare entities like MPNSTs. Collaborating with cancer registries could bolster our insights by leveraging their expansive datasets.

## 4 Conclusion

MPNST is a rare entity. It is essential to have a deep understanding of the growth patterns of MPNSTs in the context of syndromes that predispose individuals to tumors, like the Lynch syndrome. This knowledge is crucial for accurately predicting patient outcomes and developing appropriate plans for monitoring and treatment.

## Consent

A written consent was obtained from the patient to publish this case report.

## Ethical approval

Ethical approval was deemed unnecessary by our institutional ethical committee, as the paper is reporting a single case that emerged during normal practice.

## Funding

No funds, grants, or other support were received.

## CRediT authorship contribution statement

Anis Hasnaoui: Conceptualization, Writing-Reviewing and Editing. Salma Kacem: writing-Original draft preparation. Imen Sassi: Data curation. Fakhreddine Ben Abdallah: Data curation. Sondes El Guesmi: Data curation, Investigation. All authors read and approved the final manuscript.

## Guarantor

Anis Hasnaoui.

## Declaration of competing interest

The authors declare that they have no competing interests.
